# A Novel Method of Simultaneous *In Situ* Decompression of Lateral Calcaneal Bulge and Subtalar Arthrodesis *Via* a Single Incision for Malunion After Calcaneal Fractures

**DOI:** 10.1111/os.12686

**Published:** 2020-05-28

**Authors:** Tao Zhang, Wei Chen, Guangrong Yu, Xuebin Zhang, Yingze Zhang

**Affiliations:** ^1^ Department of Orthopaedic Surgery The Third Hospital of Hebei Medical University Shijiazhuang China; ^2^ Department of Orthopaedic Surgery Tongji Hospital of Tongji University Shanghai China

**Keywords:** Calcaneal fractures, Malunion, Minimally invasive techniques, Subtalar arthrodesis

## Abstract

**Objective:**

The aim of this study was to introduce a novel method of simultaneous *in situ* decompression of lateral calcaneal bulge and subtalar arthrodesis via a single incision for malunion after calcaneal fractures and evaluate the feasibility of this method.

**Methods:**

From September 2010 to October 2011, six patients (five males and one female) with malunion and delayed heel pain after conservative treatment of displaced intra‐articular calcaneal fractures were included in our study. The mean age of the six patients was 32.9 years (range, 25–71 years). Patients were treated with this novel technique at our department and the functional outcomes were assessed using the American Orthopaedic Foot and Ankle Society (AOFAS) scores during follow‐up. Information of the six patients including surgical data and pre/postoperative function scores were retrospectively analyzed using SPSS 19.0 statistical software.

**Results:**

The average operation time between wire insertion and incision suture was 42.2 ± 11.5 min (range, 25–56 min). The blood loss in all patients was all less than 50 ml each. The average fluoroscopy time was 25.7 ± 11.6 s (range, 11–43 s). No wound‐related and other short‐term complications were recorded. Six patients who were included in our study were followed for an average period of 66.2 ± 4.7 months (range, 60–73 months). There was no patient lost to follow up. Heel pain was observed to be greatly improved preoperatively in all of the six patients. All patients restored to normal activity of life after surgery. Radiological evidence of fusion was observed in five patients. The average fusion time of these five patients was 3.5 months (range, 2–4 months). The remaining one failed to achieve fusion and the hardware removal was performed due to screw tail irritation. This patient was satisfied with the final outcomes subjectively after removal of hardware. The mean AOFAS scores at 24 months postoperative were 82.0 ± 7.0, which was greatly improved compared to preoperative (44.8 ± 10.7) (*P* < 0.05). The preoperative VAS pain scores were decreased from 5.8 ± 1.5 to 2.6 ± 1.4 at 24 months postoperative (*P* < 0.05) and slightly decreased to 2.0 ± 1.7 at 48 months postoperative (*P* < 0.05). No surgery‐related complications were observed in any of the patients.

**Conclusions:**

The novel technique can effectively relieve the heel pain, prompt functional recovery, decrease the incidence of complications, simplify the surgical procedure, and shorten the learning curve. Therefore, the technique is a feasible and worthwhile alternative in treating malunion after calcaneal fractures.

## Introduction

Calcaneal fractures can lead to malunion due to failure of the conservative treatment or surgery, which usually results in shortening and widening of calcaneus associated with severe varus or valgus deformity occasionally^1^, ^2^. Intractable pain is believed to be one of the main causes of patients being incapable of returning to work following calcaneal malunion^3^. The sources of pain following calcaneal malunion may be complicated. Subtalar or calcaneocuboid joint arthritis, irritation of lateral calcaneal bulge on peroneal tendon, and anterior or posterior ankle impingement caused by morphological changes of the calcaneus are all possible sources of pain. Thus, how to accurately diagnose the source of heel pain following calcaneal malunion and perform targeted treatment poses a major challenge for clinicians^4^. Currently, subtalar arthrodesis or triple arthrodesis are the most frequently used methods for intractable hindfoot pain due to causes such as subtalar arthritis following calcaneal fractures^5^, ^6^, ^7^. However, due to the characteristics of the blood supply to the surrounding soft tissues of the calcaneus and poor soft tissue envelope, arthrodesis via an extended lateral approach may be associated with high incidence of incision‐related complications^8^. Thus, a large number of scholars began to treat malunion following calcaneal fractures by minimally invasive approaches, which has achieved satisfactory results. Some of the minimally invasive techniques can indeed reduce the incidence of wound‐related complications and the difficulty of surgery; they can also shorten the learning curve^9^, ^10^. Although minimally invasive techniques can effectively reduce the incidence of complications, most of them can only achieve *in situ* subtalar arthrodesis regardless of the complexity of pain. However, this technique remains incapable of taking into consideration if there simultaneously exists lateral calcaneal bulge, varus or valgus deformity, and calcaneal shortening^9^, ^10^, ^11^. To broaden the indications of minimally invasive subtalar arthrodesis techniques, we designed a novel method of simultaneous *in situ* decompression of lateral calcaneal bulge and subtalar arthrodesis via a single incision. The technique maximally reduced the irritation to soft tissue and decreased the surgical difficulty. The aims of this study were: (i) to introduce a novel minimally invasive technique which has wider indications; (ii) to evaluate the feasibility of this novel method in treating malunion after calcaneal fractures; and (iii) to assess the effectiveness of the new technique.

## Patients and Methods

From September 2010 to October 2011, six patients with delayed heel pain after conservative treatment of displaced intra‐articular calcaneal fractures were treated with our technique at our department. Inclusion criteria were as follows: (i) malunion after calcaneal fractures; (ii) Stephens and Sanders I or II type; and (iii) presented with intractable heel pain affecting normal life. Exclusion criteria were as follows: (i) concomitant with other severe limb dysfunction affecting prognosis; (ii) concomitant with severe underlying disease such as diabetes; and (iii) concomitant with severe calcaneal deformities such severe shortening, varus and valgus deformity, or loss of height. Malunion and broadening of the calcaneus can be seen on the axial view radiographs for all the six cases. The patients consisted of five males and one female. The mean age was 32.9 years (range, 25–71 years). Among these patients, there was one case of type I and five cases of type II, according to the classification of Stephens and Sanders^12^. The sources of pain were identified via the diagnostic anesthesia with 1% lidocaine^4^, ^13^. The sources of pain originate from the irritation of broadened lateral calcaneal bulge on the peroneal tendon sheath in one case (type I) and both the post‐traumatic subtalar arthritis and irritation of lateral calcaneal bulging on peroneal tendon sheath in the other five cases (Sanders type II). The study was approved by the ethics committee of the Third Hospital of Hebei Medical University and conducted in accordance with the Declaration of Helsinki. Signed informed consent was obtained from each patient.

### *Surgical Procedures*

The surgeries consisted of two parts: *in situ* decompression of the lateral bulged calcaneal wall and subtalar arthrodesis. Surgical technique was selected alone or in combination according to the causes of pain of each patient. *In situ* decompression of the bulged calcaneal wall was employed to release the irrigation of lateral calcaneal bulge on peroneal tendon sheath. Subtalar arthrodesis was employed to relieve pain secondary to post‐traumatic subtalar arthritis. Both minimally invasive techniques were performed through the same longitudinal single 3–4 cm incision.

### *In situ* Decompression of Lateral Calcaneal Bulge

#### *Step 1. Anesthesia and Position*

The operative procedures were performed under epidural anesthesia. Patients were placed in the lateral decubitus position on a radiolucent operating table with the affected side upward. The location and range of lateral bulge on the lateral wall was determined under fluoroscopic visualization and marked.

#### *Step 2. Localization and In Situ Decompression*

**Figure 1:**
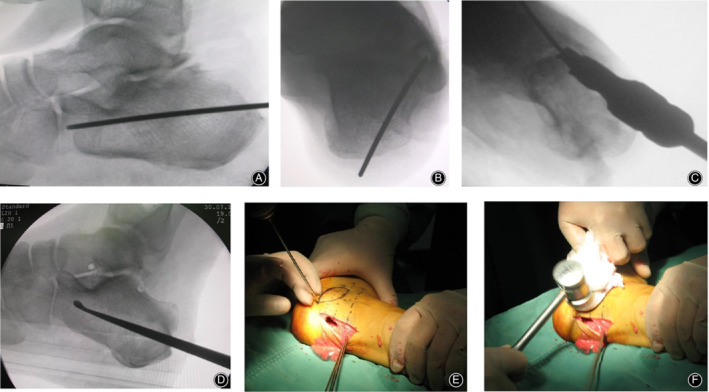
(A, B) A 2‐mm Kirschenr wire was inserted as a guide wire into the site of 5–7mm beneath the cortex of the later calcaneal bulge in parallel to the cortex. (C) 8‐mm core drill was used to create the bone tunnel along the guide wire (D) Curette was used to scrape the residual cancellous bone beneath the lateral calcaneal bulge (E) Kirschenr wire was used to create several holes along the border of the lateral calcaneal bulge perpendicular to the cortex (F) Hammer was used to impact the bulge into the cavity.

A 2 cm longitudinal incision was made at the lateral calcaneal bulge parallel to the Achilles tendon. Care should be taken to protect the sural nerve. A 2‐mm Kirschner wire was inserted as a guide wire into the site of 5–7 mm beneath the cortex of the later calcaneal bulge in parallel to the cortex (Fig. 1 A,B). The location of the guide wire was confirmed by X‐ray fluoroscopy. The step may require several iterations to obtain a satisfied guide wire insertion under fluoroscopic guidance. A 8‐mm core drill was used to create the bone tunnel along the guide wire beneath the bulge of the lateral calcaneal wall (Fig. 1C). A curette was used to scrape the residual cancellous bone beneath the lateral calcaneal bulge in the bone tunnel under the fluoroscopic guidance (Fig. 1D). A Kirschner wire was used to create several holes along the border of the lateral calcaneal bulge perpendicular to the cortex and extend into the inferior cavity beneath the bulge (Fig. 1E). Then a thick piece of sterile gauze is placed above the tunnel on the lateral side of the bone bulge to protect the soft tissue. A hammer was used to impact the bulge into the cavity (Fig. 1F). After confirming the complete collapse of lateral bulge into the inferior cavity under fluoroscopic guidance, the irrigation of lateral calcaneal bulge on peroneal tendon sheath could be effectively decompressed. If instability exists regarding the collapse of bulge in the cavity, a percutaneous screw with gasket can be used to impact the later wall into the cavity completely and fix it.

### *Subtalar Arthrodesis*

#### *Step 1. Anesthesia and Position*

Anesthesia and interoperative position was the same as *in situ* decompression of lateral calcaneal bulge technique.

#### *Step 2. Localization and Creating Bone Tunnel and Bone Graft Fusion*

**Figure 2:**
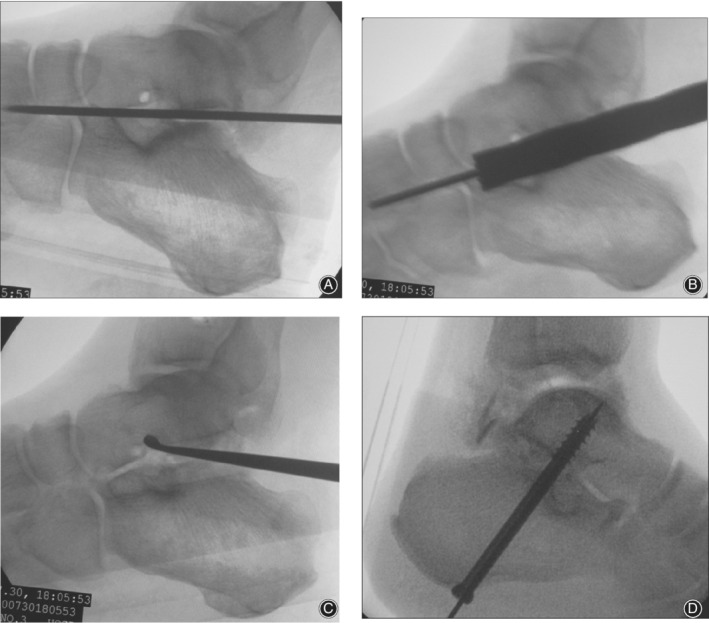
(A) Guide wire was insert to determined at the center of the subtalar joint (B) Core reamers with different diameters were used to expand the bone tunnel gradually (C) Curette was used to scrape the debris and cartilage through the tunnel as much as possible (D) Two cannulated screws were inserted along the guide wire to fix the subtalar joint.

A 2 cm longitudinal incision was made at the junction between the lateral Achilles tendon and the sural nerve. Care should be taken to protect the sural nerve. *In situ* decompression and arthrodesis can be conducted simultaneously via the same incision if the incision was extended slightly proximally (approximately 1–2 cm). A guide wire was inserted into the subtalar joint along the superior border of the calcaneal tuberosity through the incision. Under the guidance of fluoroscopy, the guide wire was determined at the center of the subtalar joint (Fig. 2A). Core reamers with different diameters were used to expand the bone tunnel gradually (Fig. 2B). A curette was used to scrape the debris and cartilage through the tunnel as much as possible (Fig. 2C). An arthroscope was introduced into the subtalar joint through the tunnel to assess the amount of fusion area obtained from the method. Then adequate cancellous bone grafts which were harvested from iliac bone and impacted into the subtalar joint were introduced via the tunnel. If *in situ* decompression and arthrodesis were conducted simultaneously, the cancellous bone harvested from the lateral calcaneal bulge can be served for bone grafts and thus bone graft harvesting from iliac can be avoided.

#### *Step 3. Internal Fixation for Subtalar Joint*

Under fluoroscopic guidance, two Kirschner wires were inserted into the planta pedis and intersected across the subtalar joint with the foot in neutral or mild valgus position. After confirming the satisfactory location under fluoroscopic guidance, two cannulated screws were inserted along the guide wire to fix the subtalar joint (Fig. 2D).

### *Postoperative Management*

If only decompression of lateral calcaneal bulge was performed, full weight‐bearing on the operated limb were allowed immediately after surgery if tolerated. If both procedures were performed, plaster immobilization was applied for 2 weeks postoperatively. At 2 weeks follow‐up, plaster was changed to removable brace boot for another 6 weeks. At 10–12 weeks postoperatively, patients were encouraged to undertake weight‐bearing exercises gradually based on X‐ray findings.

### *Follow‐Up and Outcome Evaluation*

The patients were seen for follow‐up monthly after surgery and contact was made after radiological evidence of bone fusion was obtained. Functional outcomes and complications were assessed and recorded at the time of the 24‐month follow‐up. Functional outcomes were assessed using the American Orthopaedic Foot and Ankle Society (AOFAS) scores. Pain was evaluated according to a visual analog scale (VAS).

### *American Orthopaedic Foot and Ankle Society (AOFAS) Scores*

The AOFAS score was employed to evaluate postoperative recovery of hind foot. The AOFAS score system mainly includes the three aspects of pain, function, and alignment. The maximum score of AOFAS score system is 100 points based on questionnaire and measurement (excellent: 90–100, good: 80–89, fair: 70–79, poor: <70).

### *Pain VAS Score*

The VAS is a simple measurement method for pain intensity in clinical practice. Pain was evaluated using a visual analogue scale (VAS) ruler which has a scale from 0 (no pain) to 10 (unbearable pain). A higher score on the VAS ruler suggests a higher level of pain. The clinical evaluation is divided into excellent (0–2), good (3–5), fair (6–8), and poor (>8).

### *Statistical Analysis*

All data were analyzed using SPSS 19.0 statistical software (IBM Corp., Armonk, NY, USA). The continuous variables are expressed as means ± standard deviation (SD), and significant differences were determined by Student's t‐test. A P‐value of <0.05 was considered as statistically significant.

## Results

One patient received only minimally invasive decompression of lateral calcaneal bulge and five patients received decompression of lateral calcaneal bulge and subtalar arthrodesis via a single incision simultaneously. All patients completed the follow‐up with an average period of 66.2 ± 4.7 months (range, 60–73 months) (Fig. . 3).

**Figure 3:**
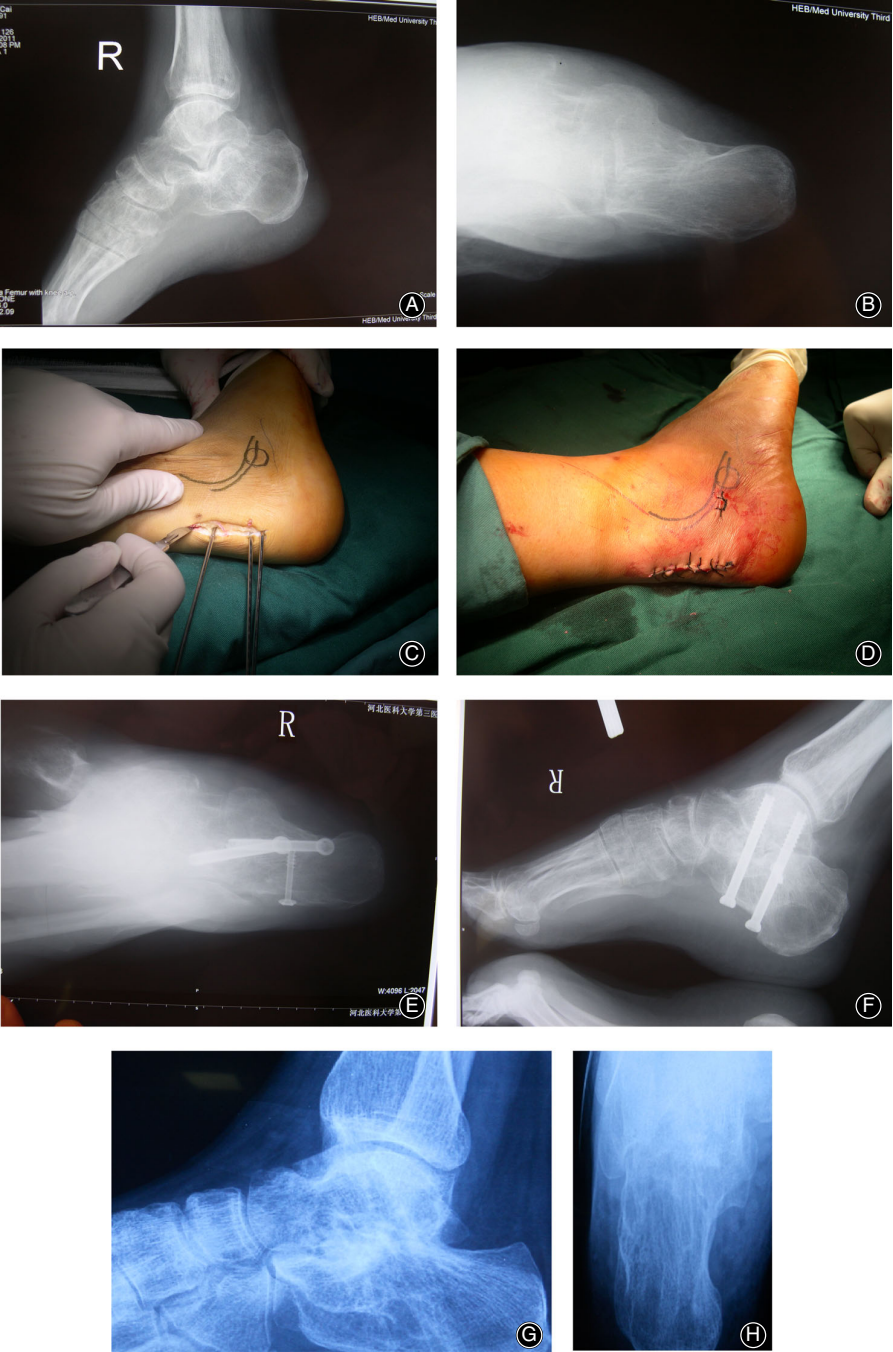
(A, B) Preoperative X ray (C, D) The two procedures were performed *via* a single 3–4 cm incision (E, F) Postoperative X ray (G, H) X ray at one year follow up after surgery, the subtalar joints achieved bony healing.

### *Surgical Information*

The average operation time between wire insertion and incision suture was 42.2 ± 11.5 min (range, 25–56 min). The blood loss in all patients was all less than 50 ml each. The average fluoroscopy time was 25.7 ± 11.6 s (range, 11–43 s).

### *Clinical Improvement*

Radiological evidence of fusion was observed in five patients. The average fusion time of these five patients was 3.6 ± 0.9 months (range, 2–4 months). Preoperative heel pain was greatly improved in all patients after surgery. All patients restored to normal activity of life. The mean AOFAS scores at 24 months postoperative were 82.0 ± 7.0 (range, 72–93), which was greatly improved compared to preoperative (44.8 ± 10.7) (range, 35–63) (T = 11.9 *P* < 0.05). Three of the four subjective indexes of AOFAS including at 24 months postoperative were all greatly improved compared to the preoperative values (Table 1). The preoperative VAS pain scores were decreased from 5.8 ± 1.5 to 2.6 ± 1.4 at 24 months postoperative (T = 19.0 *P* < 0.001) and slightly decreased from 2.6 ± 1.4 to 2.0 ± 1.7 at 48 months postoperative (T = 3.2 *P* < 0.05).

**Table 1:** The preoperative and postoperative AOFAS scores of the six patients

	AOFAS Score	Pain	Limitation of activity	Maximal walking distance	Walking surface
Pre‐op	44.8 ± 10.7	6.7 ± 10.3	5.0 ± 1.5	2.7 ± 1.0	2.5 ± 1.2
Post‐op	82.0 ± 7.0	31.7 ± 4.1	8.5 ± 1.6	4.5 ± 1.2	3.3 ± 0.8
T	11.9	7.3	7.0	3.4	1.5
*P*	*P* < 0.001	*P* < 0.001	*P* < 0.001	*P* < 0.05	*P* > 0.05

### *Complications Information*

One elderly patient who has a longtime smoking history failed to achieve fusion and the hardware removal was performed due to screw tail irritation. This patient was satisfied with the final outcomes subjectively after removal of hardware. Hardware removal were ultimately performed for two of the five patients who achieved fusion at the request of the patients. No wound and soft tissue‐related complications were observed in any of the patients.

## Discussion

Intra‐articular calcaneal fractures caused by high‐energy trauma are vulnerable to subtalar arthritis and malunion despite the conservative or surgical treatments, leading to persistent postoperative pain and low patient satisfaction^14^. Pain associated with malunion can be variable in origin, such as varus or valgus deformity of the calcaneus, a loss of height, planta pedis bulge, and posterior malleolus impingement. Increased width and bulge formation around the lateral wall after calcaneal malunion will limit the available space of lateral structures (e.g., peroneal tendon, sural nerve), leading to calcaneo‐fibular impingement and irritation of the peroneal tendon. Persistent irritation may cause peroneal tendonitis and peroneal tendon dislocations or subluxation, which is also a main reason for chronic lateral ankle pain^15^. Despite the radiological evidence of malunion and arthritis, the clinical symptoms of most patients are still not directly related with radiological manifestations^16^, which will aggravate the difficulty of diagnosing the sources of pain after calcaneal malunion. Thus, scholars began to determine the sources of dysfunction of calcaneal malunion with diagnostic anesthesia with 1% lidocaine and 0.5% bupivacaine^17^. Myerson and Quill demonstrated that diagnostic anesthesia determined the sources of pain in 87.5% of patients^17^. Extended lateral approach is most commonly used to correct deformity and improve prognosis because it can provide wider surgical view. However, extended lateral approach with L‐shaped incision may cause severe soft tissue damage and increase the risk of soft tissue complications; this risk will be increased if traction and bone grafting is conducted because it can further increase the soft tissue tension^15^, ^18^. Many scholars began to try to use minimally invasive subtalar arthrodesis. Currently, *in situ* subtalar arthrodesis is one of the most commonly used methods. Scholars have tried to use an assisted arthroscopy and obtained good clinical outcomes with lower incidence of complications^19^, ^20^. However these minimally invasive techniques cannot deal with other severe deformities if they exist simultaneously, such as severe shortening and valgus deformities. Current minimally invasive techniques have narrow indications and cannot be used in various types of calcaneal malunion. In order to extend the indications of minimally invasive techniques in calcaneal malunion, maximally reduce the risk of complications, simplify the surgical difficulty, and shorten the learning curves, we designed this novel technique of simultaneous *in situ* decompression of lateral calcaneal bulge and subtalar arthrodesis via a single incision. This technique achieved satisfactory outcomes.

### *Features of the Technique*

Irritation of lateral calcaneal bulge on peroneal tendon sheath second to traumatic subtalar arthritis is a main cause of pain. Cabot lateral wall bulge after calcaneal fractures can lead to severe pain or even rupture of the tendons of peroneus longus and brevis muscles^21^. Isbister suggested that lateral ankle pain may be caused by secondary tenosynovitis resulting from the impingement of lateral calcaneal bulge after calcaneal fractures on the lateral ankle and compression on the tendon of peroneus longus and brevis muscles^22^. A radiographic study conducted by Chen *et al*. further confirmed the correlation between the irritation of lateral bulge and pain^13^. Isbister found that local resection of lateral bulge can relieve the compression on peroneal tendons and thus achieve satisfactory results^22^. In recent years, lateral (Kocher) approach or extended lateral approach with L‐shaped incision has been proven to be effective in achieving decompression of the increased width of the calcaneus, resection of lateral bulge, release of tendon adhesion, and relocation of the peroneal tendon. Braly *et al*. performed a lateral decompression (lateral calcaneal ostectomy, peroneal tendon tenolysis, relocation and reconstruction) in 11 patients who had irritation of lateral calcaneal bulge on peroneal tendon sheath and achieved satisfactory outcomes after long‐term follow‐up.^23^ However, there have been few reports regarding how to settle the fusion of subtalar joint and relieve the compression of lateral bulge simultaneously. Some scholars proposed minimally invasive resection of lateral bulge but did not describe the surgical procedures in detail^10^. In this study, we adopted a minimally invasive technique to excavate the cancellous bone beneath the lateral bulge and thus achieved *in situ* collapse of lateral bulge. This can effectively relieve the compression, protect the cortex, and restore the normal course of peroneus muscle. It did not damage the peroneal retinaculum and thus the incidence of dislocation was avoided. Meanwhile, it reduces or avoids the iliac bone grafting because subtalar fusion can be obtained with the harvested cancellous bone. In addition, it reduced the surgical trauma and the blood loss. More importantly, this technique did not dissect the soft tissue and thus maximally avoided the incidence of soft tissue‐related complications. Posterior ankle impingement second to calcaneal malunion is also considered to a very common reason for pain^24^. In our procedures, we used a stepped drill to create the tunnel posteriorly. This process can relieve the posterior ankle impingement to some extent, which shows the special advantage of our technique.

Minimally invasive subtalar arthrodesis has become an increasingly popular topic. However, it is only available for *in situ* subtalar arthrodesis and cannot deal with other associated deformities^9^, ^11^, ^20^. Subtalar arthrodesis can effectively reduce the incidence of surgical‐related complications, surgical difficulty, and surgical time, but the assistance of arthroscopy is usually needed for early minimally invasive fusion due to the uncertainty of the fusion area. To further reduce the surgical difficulty and simplify the procedure, scholars began to perform subtalar arthrodesis without arthroscopic assistance and achieved satisfactory results^25^. Mann *et al*. suggested that 50% fusion area can achieve satisfactory results^26^. Thus, minimally invasive technique has been proven to be one of the effective subtalar arthrodesis and the only question is its limited indication. In order to ensure the fusion objectives, we used the arthroscopy to determine the bone bed. One elderly patient who failed to achieve fusion had a long history of smoking; smoking and advanced age are both predictive factors for fusion failure^27^. This patient was satisfied with the final outcomes which may be due to the pain relief resulting from the successful decompression of later calcaneal bulge. In addition, the joint space would be increased if articular surfaces of the subtalar joint were completely removed and thus ecstrophy can be performed as much as possible to help correct varus deformity during screw insertion.

### *Functional Outcomes*

In this study, the mean AOFAS scores at 24 months postoperatively were 82.0 ± 7.0, which was greatly improved compared to preoperative scores (44.8 ± 10.7) (*P* < 0.05). Subjectively, all patients achieved satisfactory results. During the two‐year follow‐up, one patient reported complete disappearance of pain and five patients reported occasional pain but all were greatly improved compared to preoperative state. These may be the main reasons for patients' satisfaction with the therapeutic results. Among the six patients, three had no limitation of daily activities and three had limited daily and recreational activities but crutches were not needed. Postoperative results regarding the maximum walking distance and walking surfaces were all greatly improved compared to preoperative state. Overall, the improvement of above four rewarding indexes may significantly improve the patients' satisfaction. In addition, the changes of VAS scores further confirmed this viewpoint.

Minimally invasive technique can protect the soft tissue maximally and reduce the incidence of surgery‐related complications. In our procedure, a longitudinal incision was made at the anterior border of the Achilles's tendon and a stepped drill was used to create the tunnel. It not only protected the muscle tendon and sural nerve, but also maximally protected the vulnerable soft tissue around the lateral calcaneal wall. In this study, all six patients achieved incision healing within 10–12 days postoperatively. No soft tissue‐related complications were noted. These findings confirm the efficacy of our procedure. The average operation time was only 42.2 min (range, 25–56 min) and the blood loss in all patients were all less than 50 ml each. The shortening of the operation time and reduction of blood loss may also contribute in avoiding the incidence of complications to some extent^28^. Particular shaped grafts harvested from iliac bone were not needed in our procedure, which reduced the surgical difficulty to some extent and blood loss. Meanwhile, the average fluoroscopy time is 25.7 s (range, 11–43 s), which suggests that our procedure is simple and feasible with an easy learning curve and thus worth being popularized.

### *Limitations*

This study has several limitations. Firstly, the insufficient sample size may lead to weakening of statistical characteristics. Ongoing clinical research with appropriate sample size is necessary to further confirm the efficiency of our technique. In addition, a well‐designed study should be held to investigate the relationship between various risk factors and postoperative outcomes which can further confirm the efficacy of our technique in the future. Furthermore, our surgical procedure extended the scope of application of minimally invasive technique, where *in situ* decompression of lateral calcaneal bulge and subtalar arthrodesis can be conducted simultaneously via a single incision. However, it cannot effectively deal with severe calcaneal malunion, shortening, and decreased height of deformity. Thus, the procedure is still needed to be improved to extend the scope of application.

### *Conclusions*

Our technique is successful in treating malunion after calcaneal fractures, which can effectively relieve the heel pain, prompt functional recovery, and decrease the incidence of complications. In addition, it simplified the surgical procedure, shortened the learning curve, and thus seems to be a promising option in treating malunion after calcaneal fractures.

## Supporting information


**Video S1** Surgical diagram 1: (A, B) a 2‐mm Kirschner wire was inserted as a guide wire into the site of 5–7mm beneath the cortex of the later calcaneal bulge in parallel to the cortex; (C) 8‐mm core drill was used to create the bone tunnel along the guide wire; (D) curette was used to scrape the residual cancellous bone beneath the lateral calcaneal bulge; (E) Kirschner wire was used to create several holes along the border of the lateral calcaneal bulge perpendicular to the cortex; (F) hammer was used to impact the bulge into the cavity.**Video S2** Surgical diagram 2: (A) guide wire was inserted to determine the center of the subtalar joint; (B) core reamers with different diameters were used to expand the bone tunnel gradually; (C) curette was used to scrape the debris and cartilage through the tunnel as much as possible; (D) two cannulated screws were inserted along the guide wire to fix the subtalar joint.
